# Association of diet in nurseries and physical activity with zBMI in 2–4-year olds in England: a cross-sectional study

**DOI:** 10.1186/s12889-018-6138-6

**Published:** 2018-11-14

**Authors:** Vanessa Er, Kaiseree Ioni Dias, Angeliki Papadaki, James White, Sian Wells, Dianne Stanton Ward, Chris Metcalfe, Russell Jago, Ruth Kipping

**Affiliations:** 1 Population Health Sciences, Bristol Medical School, University of Bristol, Canynge Hall, 39 Whatley Road, Bristol, BS8 2PS UK; 2 Centre for Exercise, Nutrition and Health Sciences, School for Policy Studies, University of Bristol, 8 Priory Road, Bristol, BS8 1TZ UK; 3 Centre for Trials Research, Cardiff University, 7th Floor, Neuadd Meirionnydd, Heath Park, Cardiff, CF144YS UK; 4 Department of Nutrition, Gillings School of Global Public Health, University of North Carolina at Chapel Hill, 135 Dauer Drive, Chapel Hill, NC 27599 USA; 5 National Institute for Health Research (NIHR) Collaboration for Leadership in Applied Health Research and Care West (CLAHRC West) at University Hospitals Bristol NHS Foundation Trust, 9th Floor, Whitefriars, Lewins Mead, Bristol, BS1 2NT UK

**Keywords:** Cross-sectional, Diet, Physical activity, Preschoolers, BMI, Nurseries

## Abstract

**Background:**

Childhood obesity tracks into adulthood with detrimental effects on health. We aimed to examine the relationships of diet in childcare settings and daily physical activity (PA) of preschoolers with body mass index z-score (z-BMI).

**Methods:**

We conducted a cross-sectional study of 150 children aged 2–4-years participating in the Nutrition and Physical Activity Self-Assessment for Child Care (NAP SACC) UK study to examine the associations of their diet in childcare settings and daily PA with z-BMI. Dietary intake was observed and recorded by fieldworkers using a validated tick-list food questionnaire and diet quality was assessed based on adherence to Children’s Food Trust (CFT) guidelines. PA was measured using accelerometers. We derived z-BMI scores using the UK 1990 and International Obesity Taskforce growth reference charts. Multilevel regression models were used to estimate associations between diet and PA with z-BMI separately, adjusted for age, gender, ethnicity, parental education level and clustering.

**Results:**

Among children who consumed one main meal or snack at childcare, 34.4% and 74.3% met the standards on fruits and vegetables and high sugar or fat snacks, respectively. Adherence to CFT guidelines was not associated with zBMI. Only 11.4% of children met recommended UK guidelines of three hours per day of physical activity. Minutes spent in light PA (β = 0.08, 95% CI = 0.01, 0.15) and active time (β = 0.07, 95% CI = 0.01, 0.12) were positively associated with UK 1990 zBMI scores.

**Conclusions:**

The low proportion of children meeting the standards on fruits and vegetables and high sugar or fat snacks and recommended physical activity levels highlight the need for more work to support nurseries and parents to improve preschool children’s diet and activity. In our exploratory analyses, we found children with higher zBMI were more physically active which could be attributed to fat-free mass or chance finding and so requires replication in a larger study.

**Trial registration:**

ISRCTN16287377. Registered 12 June 2014.

**Electronic supplementary material:**

The online version of this article (10.1186/s12889-018-6138-6) contains supplementary material, which is available to authorized users.

## Background

In England, 22.6% of children starting primary school are overweight or obese [1]. There is strong evidence that childhood obesity tracks into adulthood [2, 3] with detrimental effects on psychosocial health [4] and increased risk of chronic diseases in later life, including cardiovascular diseases, diabetes and certain cancers [5]. There is increasing recognition that childcare settings can play an important role in obesity prevention [6]. Since 2010, parents of 3–4 year olds are entitled to up to 15 h a week of free childcare, and in 2013 this was extended to parents of disadvantaged 2 year olds who receive certain welfare benefits. Around 71% of 2 year olds and 95% of 3–4 year olds in England attend some form of government-funded early years education, of which 38% attend day care outside of school settings [7]. Children in England are spending more time in childcare since the government increased funded childcare for 3–4 year olds from 15 to 30 h a week in September 2017. A recent survey revealed that 78% of parents took up the 30 h of childcare [8].

Studies on nutrition provision in childcare settings in the US, New Zealand and Australia suggest there is poor adherence to national nutritional guidelines for children in early years settings [9–11]. In England, a survey of 851 nurseries found that 99.3% reported serving foods and beverages consistent with the voluntary national guidelines [12], however few childcare providers met guidelines on oily fish (28.4%), vegetables (69.8%) and sugary drinks (16.8%). This was supported by findings from a survey of 130 nurseries in Liverpool, England, where guidelines on oily fish and salt were met by 20% and 15% of nurseries, respectively [13]. There is no published study on the dietary intake of preschoolers in childcare settings in the UK. Of the available studies conducted in the US [14, 15] and the Netherlands [16, 17], children consumed an insufficient amount of fruits, vegetables, and whole grains, but had excessive fat, sugar and energy intake [14–17]. In several [14, 18] but not all studies [17], children had higher vegetable intake and lower consumption of sugar-rich foods while in childcare settings than at home. However, dietary intake was often self-reported by nursery staff and parents, and thus may be subject to reporting bias.

Findings from the few studies that examined the relationship between diet and body mass index z-score (zBMI) among preschoolers are inconsistent, and none were conducted in the UK. A cross-sectional study of 2287 Greek children aged 2–5 years found that better adherence to diet and lifestyle recommendations was associated with lower odds of being overweight or obese (OR per 1 unit increment in score: 0.97, 95% confidence interval (CI): 0.95, 0.99) [19]. This was supported by a US study of 1521 preschoolers whose diet quality was assessed by adherence to the children’s Diet Quality Index (DQI) [20], but differed from the ToyBox European study, which found no relationship between diet quality and body weight status among 7063 preschoolers across six European countries (mean score for healthy weight: 68.4 vs.overweight/obese: 67.8, F = 2.71) [21]. The inconsistent findings could be due to differences in dietary measures used in these studies; weighed food records and self-reported 24 h food diary in the Greek study, 24 h dietary recall in the US study, and self-administered food frequency questionnaire (diet in the past 12 months) in the ToyBox study.

Two cross-sectional studies in the UK, where children aged 3 to 4 years were recruited from preschools in Cambridgeshire [22] and the Southampton Women’s Survey [23], found that 100% of the children met the current UK recommended physical activity (active time) guidelines of ≥180 min a day [24]. While an Australian [25] and a Canadian [26] study found that 5.1% and 83.8% of children met the guidelines of ≥180 min a day of physical activity, respectively. Additionally, Australian [27] and Canadian [28] guidelines specify that ≥60 min of the recommended daily guidelines for 3-5 year olds should be spent in moderate-to-vigorous physical activity (MVPA), where 13.7% of the 5 year olds in the Canadian [26] study met this recommendation. It is possible that children in the UK are more active than those in Australia and Canada, but variations in accelerometers, accelerometry cut-points and populations between studies preclude meaningful comparisons [29, 30].

In one of the UK-based studies, children who attended preschool full-time (≥30 h per week) spent slightly more time in MVPA than those attending part-time [23]. The other UK study found that both boys and girls were less sedentary and more active in part-time and full-time care compared to no care [22]. Several cross-sectional studies have found weak or little evidence of an association between physical activity measures and zBMI [31–35]. A US cross-sectional study of 2–5 year olds found that time spent in vigorous (OR = 0.94, 95% CI = 0.88, 1.00) and very vigorous (OR = 0.68, 95% CI = 0.48, 0.96) physical activity was inversely associated with overweight [36], but the sample size was small (*n* = 56).

To our knowledge, there is no published study on the dietary intake of UK preschoolers in childcare settings or its relationship with zBMI. Dietary intake was often self-reported by nursery staff and parents, and thus subject to reporting bias. There is limited literature looking at the number of preschool age children meeting recommended UK PA guidelines. Furthermore, the evidence on the association between PA measures and childcare attendence or zBMI is limited and inconsistent. The aim of our current study was to examine the relationship of dietary intake with zBMI, and physical activity with zBMI in preschool age children taking part in the Nutrition and Physical Activity Self-Assessment for Child Care (NAP SACC) UK study.

## Methods

### Study population

**Fig. 1:**
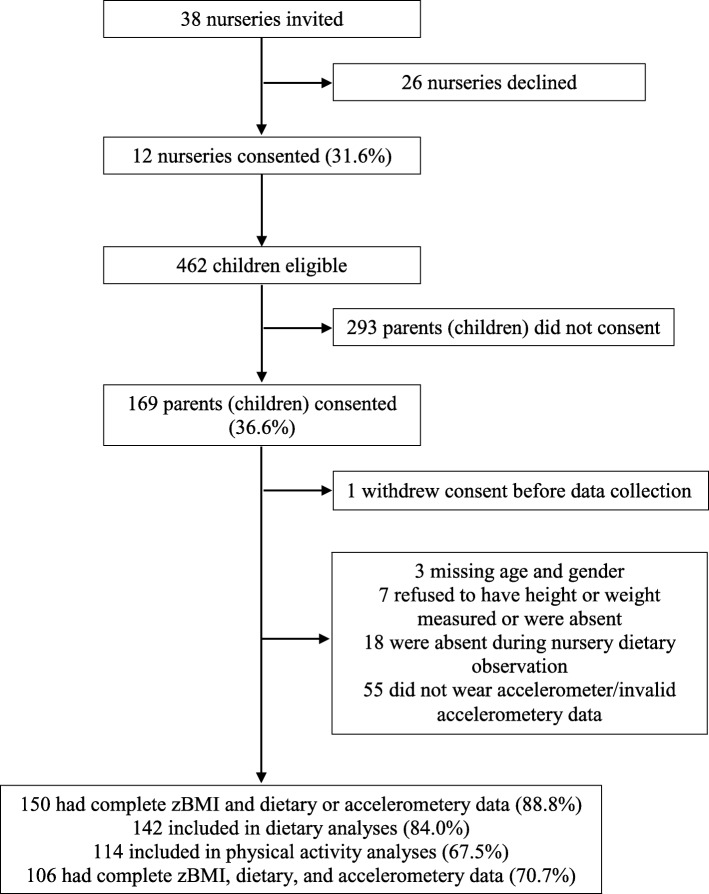
Flow diagram of participants

Children included in this study were participants in the NAP SACC UK study [37]. NAP SACC UK is a feasibility cluster randomised controlled trial to increase physical activity and healthy eating in children aged 2–4 years in nurseries and at home. Children were eligible if they attended childcare for an average of 12 h/week across the academic year September 2015–August 2016 (or 15 h/week term time only), and were provided with at least one main meal per week by the childcare setting. Between July and September 2015, 38 nurseries in North Somerset and Gloucestershire were invited to take part (Fig. 1). Of these, 12 nurseries were recruited, where 169 out of 462 parents (children) provided written consent for their children to participate in the study, but one participant withdrew consent before data collection. We excluded children with missing age and gender data (*n* = 3), who did not have their height or weight measured (*n* = 7), were absent during nursery dietary observation (*n* = 18), or were not given an accelerometer (*n* = 22) or had invalid accelerometry data (*n* = 33). This left 150 children who had complete zBMI and dietary or accelerometry data at baseline data collection for analyses.

### Data collection

At recruitment and prior to randomisation, parents completed a questionnaire on age, ethnicity, education level and household size. They were also asked to estimate the number of days and hours in a week they planned for their child to attend childcare in the academic year September 2015–August 2016.

### Anthropometry

Height (to the nearest 0.1 cm) and weight (to the nearest 0.1 kg) were measured without shoes and in light clothing using a portable SECA Leicester stadiometer, and SECA digital scales by trained fieldworkers and BMI (kg/m^2^) was derived. We estimated age- and sex-adjusted BMI (zBMI) and defined body weight categories based on the UK 1990 growth reference chart [38] and International Obesity Taskforce (IOTF) cut-offs [39] using LMS Growth Microsoft Excel add-in [40] and the zanthro module in Stata (v14.2). Weight status was categorised as follows: (UK 1990: > 2nd and < 85th centile for healthy weight vs. ≥ 85th centile for overweight/obese; IOTF: > 3rd and < 90.5 centile (boys) and > 3.7 and < 89.3 centile (girls) for healthy weight vs. ≥ 90.5 centile (boys) and ≥ 89.3 centile (girls) for overweight/obese) [38, 39]. Underweight children (UK 1990: ≤ 2nd centile; IOTF: ≤ 3rd centile (boys) and ≤ 3.7 centile (girls)) were excluded from weight category analyses.

### Diet

Children’s dietary intake at nurseries was assessed using the Child and Diet Evaluation Tool (CADET), a tick list questionnaire for young children in the UK, which has been validated against a semi-weighed food diary [41]. Trained fieldworkers completed the CADET while observing food and drink intake of each participating child during meals. Each fieldworker recorded dietary intake of up to five children. The CADET only required a tick for the item consumed in the relevant boxes - as long as the child has consumed a mouthful - with no need to record its quantity or weight. We estimated intake of four food groups: starchy foods; fruit and vegetables; non-dairy protein sources (meat, fish, and alternatives); milk and dairy foods. Additional file 1: Table S1 contains a list of food items included in each food group**.**

We assessed children’s diet quality based on their adherence to the Children’s Food Trust (CFT) guidelines. These are nationally recognised voluntary food and drink guidelines for early year settings in the UK [42], which are underpinned by a nutrient framework [43] and encompass four food groups on which to base meals and snacks: starchy foods; fruit and vegetables; non-dairy protein sources (meat, fish, and alternatives); milk and dairy foods, with additional guidance on desserts, pudding, and cakes, drinks, fat, salt, and sugar. We operationalised these guidelines (herein known as NAP SACC UK Nutrition Best Practice Standards) for main meals and snacks separately, based on the number of main meals and snacks children consumed on the day of observation (Table 2). Meals were analysed separately as the CFT have specific guidelines for main meals and snacks respectively and the participants’ nursery attendance for main meals and snacks varied. Main meals included breakfast, lunch and evening tea (dinner), and snacks included morning and afternoon snacks. We assigned children a score of 1 if they met the standard and 0 if they did not. The scores were summed to derive an overall NAP SACC UK Nutrition Best Practice Standards score (see Table 2 for details), which ranged from 0 to 8 for one main meal, 0–9 for two main meals, 0–3 for one snack, and 0–5 for two snacks. For analyses on main meal intake, we excluded children who did not consume any main meals (*n* = 12), or had breakfast only (*n* = 3). Children who did not consume any snacks (*n* = 18) were excluded from analyses on snack intake.

### Physical activity

Physical activity was objectively measured using ActiGraph GT1M accelerometers (Actigraph LLC, Pensacola, Florida, USA). The accelerometers were attached to elasticated belts which were positioned above the child’s right hip. Children were instructed to wear accelerometers for 7 days including non-nursery days. The accelerometers were set to record at 10 s epochs. Periods of ≥60 min with zero counts, allowing for 2 min of interruption [44], were taken as time the accelerometer was not worn. A day was considered valid if there were 8 h of data recorded after removing non-wear time. For physical activity analyses, only children who had at least two valid days of accelerometery data were included in the analyses (*n* = 114). We opted for a 2 day inclusion criteria to maximise the sample size from the NAP SACC UK feasibility study. Accelerometer counts per minute (cpm) were calculated by dividing the total counts by the total time after removing non-wear time. The thresholds for activity intensities were defined using criteria described in part by Puyau [45] as: sedentary (< 800 cpm), active time (≥800 cpm), light (LPA, 800 < 3200 cpm) and moderate-to-vigorous (MVPA, 3200 < 11,715 cpm) physical activity. A count value ≥11,715 cpm has been deemed as extremely high in previous literature and therefore our data were capped to this value [46].

### Statistical analyses

We computed descriptive statistics for dietary intake, adherence to the NAP SACC UK Nutrition Best Practice Standards, and physical activity levels. We used multilevel linear regression models (children were nested in nurseries, mean = 12.5, standard deviation (SD) = 4.4) to estimate the association between the NAP SACC UK Nutrition Best Practice Standards score or physical activity and zBMI, accounting for the clustering of children within the same nurseries. We repeated the analyses using multilevel logistic regression models, with weight as a categorical outcome to estimate the odds of overweight/obesity. We conducted exploratory sub-group analyses to examine physical activity levels by gender and nursery and non-nursery day separately, using multilevel linear regression models. All statistical analyses were adjusted for age, gender, ethnicity, parental education level and clustering and performed using Stata v14 (StataCorp, College Station, TX USA).

## Results

### Participant characteristics

Most of the children were 2 or 3 years old (84.9%) and of White ethnicity (88.0%) (Table 1). The gender ratio was approximately 1:1, and 70.7% had a parent with a Bachelors or higher degree. Children’s attendance at nursery was an average of 3.2 days and 25.1 h per week. Around 71% of the children were of healthy weight; with a mean zBMI of 0.45 (SD = 0.97) and 0.52 (SD = 0.95) based on the UK 1990 and IOTF reference populations, respectively.

**Table 1:** Baseline characteristics of participants

Characteristics	*n* = 150	% or Mean (SD)
Age		
2	66	44.0
3	60	40.0
4	24	16.0
Gender		
Male	72	48.0
Ethnicity		
White	132	88.0
Parental educational level		
Up to GCSEs/GCEs/O levels or similar	13	8.7
A levels/NVQs/GNVQs	31	20.7
First degree/diploma/HNC/HND	67	44.7
Higher degree (e.g. MSc, PhD)	39	26.0
Area-level deprivation (nursery)		
1 (least deprived)	62	41.3
2	75	50.0
3 (most deprived)	13	8.7
BMI category (UK 1990)		
Underweight	2	1.3
Healthy weight	106	70.7
Overweight	27	18.0
Obese	15	10.0
Height (cm)	150	96.3 (6.1)
Weight (kg)	150	15.5 (2.2)
UK 1990 zBMI	150	0.45 (0.97)
IOTF zBMI	150	0.52 (0.95)
Attendance at nursery (days/week)	149	3.2 (1.0)
Attendance at nursery (hours/week)	145	25.1 (10.7)

*UK 1990* UK 1990 growth reference chart, *IOTF* International Obesity Task Force growth reference chart

### Diet

For children who had one main meal, a large proportion of children met the standards for starchy food (> 80%), desserts, puddings, cakes (> 79%) and sugary drink (about 98%) but only around 60% of children met the guidelines for not consuming processed meat and fish products (Table 2). Among children who had two or more main meals, over half (51.5%) did not have three types of starchy food (refer to Additional file 1 for definition). Only 34.4% of children who consumed one main meal had a portion of fruit and a portion of vegetable, increasing to 70% for children eating two or more main meals. Overall adherence to the NAP SACC UK Nutrition Best Practice Standards for snacks was high, with over 90% of children consuming a portion of fruit or vegetable with some snacks, and not consuming dried fruit as a snack, and 89.4% and 100% of children who had one and two snacks not consuming sugary drinks, respectively, during snack time. However, 25.7% of children who had one snack consumed high-sugar or high-fat snacks, rising to 40.4% who had two snacks.

**Table 2:** Adherence to the NAP SACC UK Nutrition Best Practice Standards (measured by observed child food and drink consumption at nursery using CADET)

**Best Practice Standard (Main meals)**	**One main meal**^**a**^**,** ***n*** **= 64**	**Two or more main meals**^**a**^**,** ***n*** **= 66**
	*n* (%)	*n* (%)
Starchy food		
A portion as part of each meal	55 (85.9)	65 (98.5)
Three types over the course of the day	n/a	32 (48.5)
Processed potatoes never consumed	57 (89.1)	59 (89.4)
Fruit and vegetables		
A portion of fruit and a portion of vegetable at each meal	22 (34.4)	46 (69.7)
A variety of fruits and vegetables over the course of the day^b^	57 (89.1)	51 (77.3)
Meat, fish, eggs, beans and other non-dairy sources of protein		
A portion as part of each meal	32 (50.0)	40 (60.6)
Processed meat and fish products never consumed	40 (62.5)	43 (65.2)
Desserts, puddings and cakes		
Milk-based or fruit-based desserts	51 (79.7)	60 (90.9)
Beverages		
Did not consume sugary drink	63 (98.4)	65 (98.5)
**Best Practice Standard (Snacks)**	**One snack**^**c**^**,** ***n*** **= 74**	**Two snacks**^**c**^**,** ***n*** **= 47**
	n (%)	n (%)
Starchy food		
As part of at least one snack per day	n/a	34 (72.3)
Fruit and vegetables		
A portion of fruit or vegetable with some snacks	n/a	43 (91.5)
Did not consume dried fruit as a snack	69 (93.2)	45 (95.7)
High-sugar or high-fat snacks		
Did not consume as a snack	55 (74.3)	28 (59.6)
Beverages		
Did not consume sugary drink	74 (100.0)	42 (89.4)

*N/A* Not applicable

^a^ Main meal – lunch or tea. If participant had two main meals but one of it was breakfast, this is defined as one main meal

^b^ At least four types for those who had two or more main meals, and two types for those who had one main meal

^c^ Snack –morning or afternoon snack

The average fruit and vegetable intake was 5.2 portions per day (SD = 2.9) (Table 3). Conversely, consumption of non-dairy protein sources that are non-processed was relatively low, with a mean intake of 1 portion (SD = 0.8). There was no evidence for an association between food group intake or NAP SACC UK Nutrition Best Practice Standards score and zBMI, derived using either the UK 1990 or IOTF growth reference ranges. Similarly, food group intake or NAP SACC UK Nutrition Best Practice Standards score was not associated with BMI as a categorical outcome (Additional file 1: Table S2).

**Table 3:** Coefficient and 95% confidence interval for the association between food group intake, diet quality and z-BMI

	*n*	Mean (SD)	zBMI UK 1990		zBMI IOTF	
			Crude	Adjusted^a^	Crude	Adjusted^a^
Food groups (portion/day)						
Starchy foods	142	2.1 (1.1)	0.06 (−0.08, 0.20)	0.01 (− 0.13, 0.14)	0.06 (− 0.08, 0.20)	0.00 (− 0.13, 0.14)
Fruit and vegetable	142	5.2 (2.9)	0.03 (−0.03, 0.08)	0.02 (− 0.04, 0.07)	0.03 (− 0.03, 0.08)	0.02 (− 0.04, 0.07)
Meat, fish, eggs, beans, and non-dairy sources of protein	142	1.0 (0.8)	− 0.09 (− 0.28, 0.10)	−0.05 (− 0.24, 0.13)	−0.09 (− 0.28, 0.10)	−0.05 (− 0.23, 0.13)
Milk and dairy foods	142	2.4 (1.5)	0.06 (−0.05, 0.17)	0.03 (−0.07, 0.14)	0.06 (− 0.05, 0.16)	0.03 (− 0.07, 0.14)
NAP SACC Score (max score)						
One main meal (8.0)	64	5.9 (1.4)	−0.01 (− 0.18, 0.16)	−0.03 (− 0.20, 0.13)	−0.01 (− 0.18, 0.15)	−0.04 (− 0.20, 0.12)
Two or more main meals (9.0)	66	7.0 (1.4)	−0.01 (− 0.19, 0.16)	−0.06 (− 0.23, 0.12)	−0.01 (− 0.18, 0.16)	−0.05 (− 0.22, 0.12)
One snack* (3.0)	74	2.7 (0.6)	0.13 (− 0.29, 0.56)	0.07 (− 0.35, 0.50)	0.17 (− 0.25, 0.59)	0.05 (− 0.36, 0.47)
Two snacks (5.0)	47	4.1 (0.8)	0.34 (0.03, 0.65)	0.26 (− 0.07, 0.59)	0.34 (0.04, 0.64)	0.25 (− 0.07, 0.58)

*Max* Maximum

^a^Adjusted for age, ethnicity, parental education and cluster

### Physical activity

On average, only 11.4% of the 114 children in the physical activity analysis sample met the daily guidelines of ≥180 min. None of the children met the daily guidelines of ≥60 min in MVPA. The mean time spent in sedentary, LPA and MVPA per day was 494.75, 121.32 and 22.23 min, respectively (Table 4). The mean minutes spent in MVPA per hour was 2.01 min. The children in our sample spent an average of 10.51 more minutes per day in active time in childcare settings compared to non-childcare settings (Additional file 1: Table S3). There is some evidence that children spent 9.34 min per day more in LPA and 22.84 min per day more in sedentary time on nursery days compared to non-nursery days, but these associations were greatly attenuated when considered as a proportion of the overall time. Boys spent a greater proportion of time being active and a smaller proportion of time sedentary compared to girls, on both nursery days and non-nursery days (Additional file 1: Table S4). Likewise, on both nursery and non-nursery days, the mean cpm was higher in boys compared to girls.

**Table 4:** Overall, nursery and non-nursery day accelerometer sedentary time and physical activity overall and by gender

	Counts per minute	Minutes spent in physical activity, mean (SD)				Proportion of time spent in physical activity, mean (SD)			
		Sedentary	LPA	MVPA	Active time	Sedentary	LPA	MVPA	Active time
Overall									
Overall (*n* = 114)	567.30 (130.17)	494.75 (52.50)	121.32 (25.54)	22.23 (9.32)	143.55 (32.43)	77.47 (4.89)	19.02 (3.79)	3.50 (1.47)	22.52 (4.89)
Girls (*n* = 54)	543.04 (122.11)	500.03 (44.14)	117.49 (26.17)	20.27 (7.73)	137.76 (31.67)	78.35 (4.84)	18.45 (3.94)	3.20 (1.26)	21.65 (4.84)
Boys (*n* = 60)	589.14 (134.29)	490.00 (59.00)	124.77 (24.67)	24.00 (10.29)	148.77 (32.47)	76.69 (4.85)	19.54 (3.60)	3.77 (1.61)	23.31 (4.85)
Nursery day									
Overall (*n* = 113)	564.50 (160.25)	504.56 (63.32)	124.50 (33.36)	22.54 (12.16)	147.04 (43.17)	77.47 (6.09)	19.08 (4.64)	3.45 (1.80)	22.53 (6.09)
Girls (*n* = 53)	535.98 (132.41)	512.28 (50.13)	118.82 (28.43)	20.04 (8.54)	138.85 (35.07)	78.64 (5.18)	18.27 (4.14)	3.09 (1.33)	21.36 (5.18)
Boys (*n* = 60)	589.70 (178.67)	497.75 (72.78)	129.52 (36.69)	24.74 (14.34)	154.26 (48.37)	76.42 (6.66)	19.80 (4.97)	3.77 (2.09)	23.58 (6.66)
Non-nursery day									
Overall (*n* = 104)	566.21 (146.92)	481.07 (66.86)	115.40 (26.28)	21.50 (9.67)	136.89 (32.52)	77.70 (5.39)	18.76 (4.15)	3.54 (1.72)	22.30 (5.39)
Girls (*n* = 47)	535.65 (136.42)	485.84 (55.51)	111.03 (29.10)	19.50 (9.01)	130.53 (34.54)	78.77 (5.32)	18.03 (4.39)	3.20 (1.52)	21.23 (5.32)
Boys (*n* = 57)	591.40 (151.61)	477.15 (75.20)	119.00 (23.36)	23.14 (9.97)	142.14 (30.05)	76.83 (5.34)	19.36 (3.89)	3.82 (1.84)	23.17 (5.34)

*SD* Standard Deviation, *MVPA* Moderate to vigorous physical activity, *LPA* Light physical activity

Table 5 shows the association between physical activity and zBMI. Minutes spent in LPA (β = 0.08, 95% CI = 0.01, 0.15) and active time (β = 0.07, 95% CI = 0.01, 0.12) were positively associated with zBMI, based on the UK 1990 growth reference chart. The odds of being overweight/obese (Additional file 1: Table S5) using the UK 1990 chart was higher in more active children (OR = 1.13, 95% CI = 1.02, 1.25) and lower in children who spent a higher proportion of time sedentary (OR = 0.89, 95% CI = 0.80, 0.98). Similarly, children who spent a greater proportion of time in LPA (OR = 1.20, 95% CI = 1.05, 1.37) were more likely to be overweight/obese whereas those spending a greater proportion of time sedentary (OR = 0.87, 95% CI = 0.78, 0.97) were less likely to be overweight/obese, based on the IOTF growth reference chart. Based on both the UK 1990 and IOTF growth reference charts, an increase in cpm increased the odds of being overweight/obese [(UK 1990; OR: 1.01, 95% CI = 1.00, 1.01) and (IOTF; OR: 1.01, 95% CI = 1.00, 1.01)].

**Table 5:** Coefficient and 95% confidence interval for the association between physical activity and z-BMI

	*n*	Mean (SD)	zBMI UK 1990		zBMI IOTF	
			Crude	Adjusted^a^	Crude	Adjusted^a^
Counts per minute^b^	114	567.30 (130.17)	0.07 (−0.07, 0.20)	0.13 (−0.01, 0.27)	0.07 (− 0.07, 0.20)	0.13 (− 0.02, 0.27)
Minutes spent in sedentary^c^	114	494.75 (52.50)	−0.01 (− 0.04, 0.03)	−0.00 (− 0.04, 0.03)	−0.01 (− 0.04, 0.03)	−0.00 (− 0.04, 0.03)
Minutes spent in LPA^c^	114	121.32 (25.54)	0.05 (−0.02, 0.11)	0.08 (0.01, 0.15)	0.05 (−0.02, 0.11)	0.08 (0.01, 0.15)
Minutes spent in MVPA^c^	114	22.23 (9.32)	0.09 (−0.09, 0.28)	0.20 (− 0.01, 0.40)	0.09 (− 0.09, 0.27)	0.19 (− 0.01, 0.39)
Minutes spent in active time^c^	114	143.55 (32.43)	0.04 (−0.02, 0.09)	0.07 (0.01, 0.12)	0.04 (−0.02, 0.09)	0.06 (0.01, 0.12)
Proportion of time spent in sedentary	114	77.47 (4.89)	−0.02 (− 0.06, 0.02)	−0.04 (− 0.07, 0.00)	−0.02 (− 0.05, 0.02)	−0.03 (− 0.07, 0.00)
Proportion of time spent in LPA	114	19.02 (3.79)	0.03 (−0.02, 0.07)	0.04 (−0.00, 0.09)	0.03 (− 0.02, 0.07)	0.04 (− 0.01, 0.09)
Proportion of time spent in MVPA	114	3.50 (1.47)	0.05 (−0.07, 0.16)	0.10 (−0.03, 0.23)	0.05 (− 0.07, 0.16)	0.10 (− 0.03, 0.22)
Proportion of time spent in active time	114	22.52 (4.89)	0.02 (−0.02, 0.06)	0.04 (−0.00, 0.07)	0.02 (− 0.02, 0.05)	0.03 (− 0.00, 0.07)

^a^ Adjusted for age, ethnicity, parental education and cluster

^b^ zBMI for each 100 counts per minute increment

^c^ zBMI for each 10 min increment

## Discussion

Our study showed that while the overall diet quality was good, the proportion of children who consumed high sugar or high fat snacks while at nursery was relatively high, which is consistent with some [15, 17], but not all studies [14, 18]. Unlike most studies suggesting inadequate consumption of fruits and vegetables among preschoolers at childcare [15, 16], the average fruit and vegetable intake in our study was high. This could be due to differences in dietary assessment, as, according to the CADET [41], children in our study were considered to be consuming a food item if they had a mouthful, and were assigned the respective standard portion size, regardless of the actual amount eaten. This suggests that a tick list food diary might not be sensitive enough to detect differences in portion sizes and thus amount of food intake. Futhermore, the relatively low proportion of children who met the standard of one portion of fruit and one portion of vegetable at main meal suggests that the average fruit and vegetable intake was largely attributable to fruit consumption.

Neither food group intake nor the NAP SACC UK Nutrition Best Practice Standard score was associated with zBMI in this sample of preschoolers. This could be due to variation in the components of the dietary index and scoring criteria. A study in children aged 9–10 years found that adherence to dietary guidelines as assessed by the DQI and Healthy Eating Index (HEI), both including guidelines on nutrients, was associated with lower waist circumference and body fat, but not the Mediterranean Diet Score (MDS) [47], which is a food group-based index as the NAP SACC UK Nutrition Best Practice Standard. This was consistent with findings from the ToyBox study, which also used a food group-based index to assess diet quality and did not observe an association with obesity [21]. Furthermore, physical activity or sedentary behaviour was included as a component of the index in two studies that found an inverse association between diet quality and obesity among preschoolers [19, 20]. Adherence to dietary guidelines in earlier studies [19, 21] was assessed based on total daily dietary intake whereas in this study, we assessed dietary intake at childcare only, and separately for main meals and snacks. As a result, it precluded the operationalisation of several guidelines that relate to total daily dietary intake such as those that pertain to dairy foods, and might explain the lack of association with zBMI in our study.

It is likely that food intake at home is more important when looking at associations with zBMI, especially for children who attend childcare part-time (i.e. consume one main meal only), as several studies have reported lower vegetable and higher sugar intake when preschoolers were at home compared to that when attending childcare [14, 18]. Home environment has also been reported to have a stronger impact on preschoolers’ zBMI than early education setting (i.e.large proportion of the variation in preschoolers’ zBMI was explained by diet in the home environment) [48]. These suggest the need to assess children’s diet at home when studying associations with zBMI. Additionally, findings from several systematic reviews found that active parental enagagement is key in effective interventions for promoting healthy eating or preventing obesity in childcare settings [49, 50].

In this study, none of the children achieved ≥60 min in MVPA and only 11.4% of children met current guidelines of ≥180 min of physical activity per day, with children spending an average of 143.55 min/day in active time. This is not consistent with findings from other UK-based preschool age populations [22, 23]. This difference is likely to be a function of accelerometer cut-points, as the previous studies used a lower cut-point for determining active time levels (active time: ≥38 cpm [22], ≥20 cpm [23]); meaning a greater proportion of their samples would be meeting the guidelines than if they used the cut-points in our study. The proportion of time spent being physically active (22.5%) was greater than findings from a European study (15.3%) [51] and an Australian study (16.4%) [25], but lower than findings from a Canadian study (50%) [26].

Our study found that children spent more minutes in LPA and sedentary time on nursery days than non-nursery days, however this difference was not observed when considered as a proportion of time. Findings from another UK-based study found that children were more engaged in LPA and MVPA and spent less time sedentary when in childcare compared to at home [22]. In line with other cross-sectional studies [22, 23, 25, 31, 32], we found some evidence that boys were more active than girls in our study. The current study also highlights that this difference is observed on both nursery and non-nursery days.

We showed some evidence of an association between children with higher zBMI scores being more active and less sedentary, using both the UK 1990 and IOTF growth reference charts. Our results are consistent with studies which have found a weak positive correlation between z-BMI score and activity [32] and a positive association with MVPA [31]. Likewise, we found evidence of higher physical activity levels increasing the likelihood of being overweight/obese which contradicts findings from an American study which found that being more sedentary increased the chance of being overweight/obese by 3.6 times [36]. Differences in the results may be accounted for by the use of lower accelerometry cut-points as well as the Centers for Disease Control and Prevention (CDC) growth charts in calculating the zBMI scores.

Our results also contradict findings from several European studies [33–35, 52] which found little evidence of an association between BMI z-score and physical activity. BMI as a measure is not able to differentiate between the contributions of fat mass and fat free mass [53]; therefore the positive association between physical activity and zBMI may reflect children with a higher fat free mass being more physically active. A UK-based study [54] assessed the associations between physical activity and sedentary behaviour with body composition in 4 year olds and found that VPA was inversely associated with total and abdominal adiposity. The findings from the current study may also be explained by children with higher zBMI scores being more physically developed. Research suggests that fundamental motor skills (FMS) are associated with perceived physical competence, more advanced development, and participation in physical activities [55, 56]. A study which identified correlates of physical activity in 2 year olds found that higher levels of MVPA were observed in older children and those with normal gross motor development [52]. Findings from other studies suggest that children in the normal, and sometimes overweight groups, have better FMS than obese preschool age children [56, 57]. However, children in these study populations were aged 3–7 years old and the association between zBMI categories and FMS might differ significantly in 2–4-year olds.

### Strengths and limitations

Our study is one of the first to examine dietary intake and physical activity at childcare and their associations with zBMI in UK preschoolers. Dietary intake was assessed by trained fieldworkers and we had an objective measure of physical activity using accelerometer, thereby limiting reporting bias. Nonetheless, some studies have found percentage body fat and/or fat free mass as a better indicator of adiposity compared to zBMI [58]. It is widely known that BMI declines after the first year of life until around the age of 6 years before it rises again (adiposity rebound) [59]. Therefore, a single BMI measurement may not be senstitive enough to detect an association between diet quality and obesity in preschool age children. We observed dietary intake of children at nursery on a single day only, which may not reflect their usual intake. Parents were asked to report their children’s food intake using a home food diary but there was a low response rate and many filled in the diary on a different date from the nursery observation. Therefore, we were not able to estimate the daily dietary intake of children and compare their dietary intake by setting (nursery vs. home). Unlike dietary indices such as the HEI, DQI or the MDS, the NAP SACC UK Nutrition Best Practice Standard has not been validated and may not be suitable for examining the relationship between diet quality and zBMI. Up to 7 days of accelerometry data were recorded at 10 s epochs which provides us with reliable data. However, to maximise the number of children in our sample we analysed children with at least 2 days of valid data where ideally we would want to have more days of data to be more representative of the children’s average physical activity levels. With there being no standardised international accelerometry cut-points for preschool age children, it is difficult to draw conclusions and comparisons of physical activity levels across studies. It is also important to note that we did not specifically record naps and as such the estimates of physical activity may include time when the children were napping. Participants in our study were mainly of White ethnicity and high socioeconomic status (SES) as indicated by parental education level which thus limits the generalisability of our findings. We did not collect data on non-participants but it is plausible that children of low SES who declined to take part in our study tended to be overweight or obese and inactive, and to have poor diet quality. Therefore, the association between diet in childcare and zBMI could be biased to the null whereas the association between daily PA and zBMI could be overestimated due to self-selection in our study population. We also cannot exclude the possibility of chance findings in the context of limited power due to the small sample size and multiple testing. To minimise multiple testing, we had decided a priori on the dietary and physical activity variables to be tested and used established dietary guidelines for categorisation.

## Conclusion

In this study of 2–4-year olds in the UK attending nursery for an average of 25 h per week, we did not find an association between zBMI and the quality of diet consumed by children at nursery, but found a positive association between time spent in LPA and in active time with zBMI. Evidence on the association of activity and weight is mixed thus it should be investigated further in larger samples of preschool children in the UK. While the quality of diet consumed in nursery was reasonably good, the proportion of children meeting the standards on fruits and vegetables and high sugar or fat snacks was low and only 11.4% of children were meeting the recommended 3 h per day of physical activity. Therefore, a public health priority is to work with nurseries and parents to support them to increase preschool children’s vegetable intake and activity, and reduce consumption of high sugar or fat snacks.

## Additional file

Additional file 1: Food groups and relationships of diet and physical activity with overweight/obesity. Definition of food groups and food items, physical activity and sedentary time by nursery vs. non-nursery days, and the associations of diet and physical activity with overweight/obesity. (DOCX 23 kb)

## Abbreviations

CADET: Child and Diet Evaluation Tool
CDC: Centers for Disease Control and Prevention
CFT: Children’s Food Trust
CI: Confidence interval
cpm: Counts per minute
DQI: Diet Quality Index
FMS: Fundamental motor skills
HEI: Healthy Eating Index
IOTF: International Obesity Taskforce
LPA: Light physical activity
MDS: Mediterranean Diet Score
MVPA: Moderate-to-vigorous physical activity
NAP SACC: Nutrition and Physical Activity Self-Assessment for Child Care
PA: Physical activity
SD: Standard deviation
zBMI: Body mass index z-score

## References

[1] NHS Digital: National Child Measurement Programme: England, 2016/17 school year. 2017. https://digital.nhs.uk/services/national-child-measurement-programme/. Accessed 21 Sept 2018.

[2] Singh AS, Mulder C, Twisk JWR, van Mechelen W, Chinapaw MJM (2008). Tracking of childhood overweight into adulthood: a systematic review of the literature. Obes Rev.

[3] Simmonds M, Llewellyn A, Owen CG, Woolacott N (2016). Predicting adult obesity from childhood obesity: a systematic review and meta-analysis. Obes Rev.

[4] Puhl RM, Latner JD (2007). Stigma, obesity, and the health of the nation's children. Psychol Bull.

[5] Llewellyn A, Simmonds M, Owen CG, Woolacott N (2016). Childhood obesity as a predictor of morbidity in adulthood: a systematic review and meta-analysis. Obes Rev.

[6] Larson N, Ward DS, Neelon SB, Story M (2011). What role can child-care settings play in obesity prevention? A review of the evidence and call for research efforts. J Am Diet Assoc.

[7] Department for Education. Provision for children under five years of age in England. London: Department for Education; 2017.

[8] Department for Education. Childcare and early years survey of parents: 2017 follow-up survey. London: Department for Education; 2018.

[9] Erinosho T, Dixon LB, Young C, Brotman LM, Hayman LL (2011). Nutrition practices and children’s dietary intakes at 40 child-care centers in New York City. J Am Diet Assoc.

[10] Gerritsen S, Dean B, Morton SMB, Wall CR (2017). Do childcare menus meet nutrition guidelines? Quantity, variety and quality of food provided in New Zealand early childhood education services. Aust N Z J Public Health.

[11] Yoong SL, Skelton E, Jones J, Wolfenden L (2014). Do childcare services provide foods in line with the 2013 Australian dietary guidelines? A cross-sectional study. Aust N Z J Public Health.

[12] Neelon SEB, Burgoine T, Hesketh KR, Monsivais P (2015). Nutrition practices of nurseries in England. Comparison with national guidelines. Appetite.

[13] Parker M, Lloyd-Williams F, Weston G, Macklin J, McFadden K (2011). Nursery nutrition in Liverpool: an exploration of practice and nutritional analysis of food provided. Public Health Nutr.

[14] Sisson S, Kiger A, Anundson K, Rasbold A, Krampe M, Campbell J, DeGrace B, Hoffman L (2017). Differences in preschool-age children's dietary intake between meals consumed at childcare and at home. Prev Med Rep.

[15] Ball SC, Benjamin SE, Ward DS (2008). Dietary intakes in North Carolina child-care centers: are children meeting current recommendations?. J Am Diet Assoc.

[16] Goldbohm RA, Rubingh CM, Lanting CI, Joosten KFM (2016). Food consumption and nutrient intake by children aged 10 to 48 months attending day care in the Netherlands. Nutrients.

[17] Gubbels JS, Raaijmakers LG, Gerards SM, Kremers SP (2014). Dietary intake by Dutch 1- to 3-year-old children at childcare and at home. Nutrients.

[18] Lehtisalo J, Erkkola M, Tapanainen H, Kronberg-Kippila C, Veijola R, Knip M, Virtanen SM (2010). Food consumption and nutrient intake in day care and at home in 3-year-old Finnish children. Public Health Nutr.

[19] Manios Y, Kourlaba G, Grammatikaki E, Androutsos O, Moschonis G, Roma-Giannikou E (2010). Development of a diet-lifestyle quality index for young children and its relation to obesity: the preschoolers diet-lifestyle index. Public Health Nutr.

[20] Kranz S, Findeis JL, Shrestha SS (2008). Use of the revised Children's diet quality index to assess preschooler's diet quality, its sociodemographic predictors, and its association with body weight status. J Pediatr.

[21] Pinket AS, De Craemer M, Huybrechts I, De Bourdeaudhuij I, Deforche B, Cardon G, Androutsos O, Koletzko B, Moreno L, Socha P (2016). Diet quality in European pre-schoolers: evaluation based on diet quality indices and association with gender, socio-economic status and overweight, the ToyBox-study. Public Health Nutr.

[22] Hesketh KR, Griffin SJ, van Sluijs EMF. UK preschool-aged children's physical activity levels in childcare and at home: a cross-sectional exploration. Int J Behav Nutr Phys Act. 2015;12. 10.1186/s12966-015-0286-1 PMC4583748 26410252

[23] Hesketh KR, McMinn AM, Ekelund U, Sharp SJ, Collings PJ, Harvey NC, Godfrey KM, Inskip HM, Cooper C, van Sluijs EM (2014). Objectively measured physical activity in four-year-old British children: a cross-sectional analysis of activity patterns segmented across the day. Int J Behav Nutr Phys Act.

[24] Department of Health. Start Active, Stay Active: a report on physical activity from the four home countries’ Chief Medical Officers. London: Department of Health; 2011.

[25] Hinkley T, Salmon J, Okely AD, Crawford D, Hesketh K (2012). Preschoolers’ physical activity, screen time, and compliance with recommendations. Med Sci Sports Exerc.

[26] Colley RC, Garriguet D, Adamo KB, Carson V, Janssen I, Timmons BW, Tremblay MS (2013). Physical activity and sedentary behavior during the early years in Canada: a cross-sectional study. Int J Behav Nutr Phys Act.

[27] Department of Health. Get Up and Grow: healthy eating and physical activity for early childhood. Canberra: Australian Government; 2010.

[28] Tremblay MS, Chaput JP, Adamo KB, Aubert S, Barnes JD, Choquette L, Duggan M, Faulkner G, Goldfield GS, Gray CE (2017). Canadian 24-hour movement guidelines for the early years (0-4 years): an integration of physical activity, sedentary behaviour, and sleep. BMC Public Health.

[29] Cliff DP, Reilly JJ, Okely AD (2009). Methodological considerations in using accelerometers to assess habitual physical activity in children aged 0-5 years. J Sci Med Sport.

[30] Oliver M, Schofield GM, Kolt GS (2007). Physical activity in preschoolers: understanding prevalence and measurement issues. Sports Med.

[31] van Sluijs EM, McMinn AM, Inskip HM, Ekelund U, Godfrey KM, Harvey NC, Griffin SJ (2013). Correlates of light and moderate-to-vigorous objectively measured physical activity in four-year-old children. PLoS One.

[32] Jackson DM, Reilly JJ, Kelly LA, Montgomery C, Grant S, Paton JY (2003). Objectively measured physical activity in a representative sample of 3- to 4-year-old children. Obes Res.

[33] Vale S, Santos R, Silva P, Soares-Miranda L, Mota J (2011). Relationship of objective measurement of physical activity during school hours and BMI in preschool children. Int J Pediatr Obes.

[34] Vorwerg Y, Petroff D, Kiess W, Bluher S (2013). Physical activity in 3-6 year old children measured by SenseWear pro: direct accelerometry in the course of the week and relation to weight status, media consumption, and socioeconomic factors. PLoS One.

[35] Johansson E, Hagstromer M, Svensson V, Ek A, Forssen M, Nero H, Marcus C (2015). Objectively measured physical activity in two-year-old children - levels, patterns and correlates. Int J Behav Nutr Phys Act.

[36] Metallinos-Katsaras ES, Freedson PS, Fulton JE, Sherry B (2007). The association between an objective measure of physical activity and weight status in preschoolers. Obesity (Silver Spring).

[37] Kipping R, Jago R, Metcalfe C, White J, Papadaki A, Campbell R, Hollingworth W, Ward D, Wells S, Brockman R (2016). NAP SACC UK: protocol for a feasibility cluster randomised controlled trial in nurseries and at home to increase physical activity and healthy eating in children aged 2-4 years. BMJ Open.

[38] Cole TJ, Freeman JV, Preece MA (1995). Body mass index reference curves for the UK, 1990. Arch Dis Child.

[39] Cole TJ, Lobstein T (2012). Extended international (IOTF) body mass index cut-offs for thinness, overweight and obesity. Pediatr Obes.

[40] Pan H, Cole TJ. LMSgrowth, a Microsoft Excel add-in to access growth references based on the LMS method (version 2.77). 2012. http://www.healthforallchildren.com/shop-base/shop/software/lmsgrowth/. Accessed 7 Feb 2017.

[41] Cade JE, Frear L, Greenwood DC (2006). Assessment of diet in young children with an emphasis on fruit and vegetable intake: using CADET - child and diet evaluation tool. Public Health Nutr.

[42] Children’s Food Trust. Voluntary food and drink guidelines for early years settings in England- a practical guide. UK: Children's Food Trust; 2012.

[43] Wall C, Mucavele P, Sharp L (2012). Development and implementation of voluntary food and drink guidelines for early years settings in England. Nutr Bull.

[44] Troiano RP, Berrigan D, Dodd KW, Masse LC, Tilert T, McDowell M (2008). Physical activity in the United States measured by accelerometer. Med Sci Sports Exerc.

[45] Puyau MR, Adolph AL, Vohra FA, Butte NF (2002). Validation and calibration of physical activity monitors in children. Obes Res.

[46] Rich C, Geraci M, Griffiths L, Sera F, Dezateux C, Cortina-Borja M (2014). Quality control methods in accelerometer data processing: identifying extreme counts. PLoS One.

[47] Jennings A, Welch A, van Sluijs EMF, Griffin SJ, Cassidy A (2011). Diet quality is independently associated with weight status in children aged 9-10 years. J Nutr.

[48] Boonpleng W, Park CG, Gallo AM, Corte C, McCreary L, Bergren MD (2013). Ecological influences of early childhood obesity: a multilevel analysis. West J Nurs Res.

[49] Matwiejczyk Louisa, Mehta Kaye, Scott Jane, Tonkin Emma, Coveney John (2018). Characteristics of Effective Interventions Promoting Healthy Eating for Pre-Schoolers in Childcare Settings: An Umbrella Review. Nutrients.

[50] Sisson SB, Krampe M, Anundson K, Castle S (2016). Obesity prevention and obesogenic behavior interventions in child care: a systematic review. Prev Med.

[51] Cardon GM, De Bourdeaudhuij IM (2008). Are preschool children active enough? Objectively measured physical activity levels. Res Q Exerc Sport.

[52] Wijtzes AI, Kooijman MN, Kiefte-de Jong JC, de Vries SI, Henrichs J, Jansen W, Jaddoe VW, Hofman A, Moll HA, Raat H (2013). Correlates of physical activity in 2-year-old toddlers: the generation R study. J Pediatr.

[53] Freedman DS, Sherry B (2009). The validity of BMI as an indicator of body fatness and risk among children. Pediatrics.

[54] Collings PJ, Brage S, Ridgway CL, Harvey NC, Godfrey KM, Inskip HM, Cooper C, Wareham NJ, Ekelund U (2013). Physical activity intensity, sedentary time, and body composition in preschoolers. Am J Clin Nutr.

[55] Robinson LE (2011). The relationship between perceived physical competence and fundamental motor skills in preschool children. Child Care Health Dev.

[56] Yang SC, Lin SJ, Tsai CY (2015). Effect of sex, sge, and BMI on the development of locomotor skills and object control skills among preschool children. Percept Mot Skills.

[57] Morano M, Colella D, Caroli M (2011). Gross motor skill performance in a sample of overweight and non-overweight preschool children. Int J Pediatr Obes.

[58] Okubo H, Crozier SR, Harvey NC, Godfrey KM, Inskip HM, Cooper C, Robinson SM, Grp SS (2015). Diet quality across early childhood and adiposity at 6 years: the Southampton Women’s survey. Int J Obes.

[59] Rolland-Cachera MF, Deheeger M, Maillot M, Bellisle F (2006). Early adiposity rebound: causes and consequences for obesity in children and adults. Int J Obes.

