# Supplementary material for: Association of diet in nurseries and physical activity with zBMI in 2–4-year olds in England: a cross-sectional study
Source: BMC Public Health. 2018 Nov 14;18:1262. doi: 10.1186/s12889-018-6138-6 (PMC6236905; doi:10.1186/s12889-018-6138-6)
Supplement: Supplementary file 1 — Food groups and relationships of diet and physical activity with overweight/obesity. Definition of food groups and food items, physical activity and sedentary time by nursery vs. non-nursery days, and the associations of diet and physical activity with overweight/obesity. (DOCX 23 kb) [file 12889_2018_6138_MOESM1_ESM.docx]

**Supplementary Material**

**Supplementary Table 1.** Definition for food groups/items

| **Food item**  **(in CADET)** | **Description** |
| --- | --- |
| **Starchy foods*** | |
| C1 | Sandwich (tick filling separately). Bread, roll, toast crumpet |
| C3 | Garlic bread, naan, paratha |
| C4 | Chapatti, pitta bread, wrap, roti etc |
| C5 | Cracker, crispbread etc (NB: also included if consumed as snacks) |
| J1 | Pizza |
| J2 | Boiled rice |
| J3 | Fried rice |
| J4 | Noodles |
| J5 | Pasta-plain, cous cous |
| J6 | Pasta with tomato sauce (no meat) |
| J7 | Pasta with cheese sauce |
| J8 | Pasta with meat, fish (and sauce) |
| J9 | Yorkshire pudding, pancake |
| L1 | Boiled, mashed, jacket (potato) |
| P2 | Hi-fibre e.g. Branflakes, Weetabix, Shreddies, muesli |
| P3 | Other e.g. Cornflakes, Rice Krispies etc |
| P4 | Porridge, Ready Brek |
| *Exclude: Croissant, sweet waffles, pop tarts; Chips, roast, potato faces etc; Sugar-coated cereal e.g. Frosties, Sugar Puffs | |
| **Processed potatoes** | |
| L2 | Chips, roast, potato faces etc |
| **Fruit and Vegetables** | |
| K1 | Mixed vegetables |
| K2 | Tomatoes |
| K3 | Cucumber |
| K4 | Coleslaw |
| K5 | Other salad vegetables e.g. lettuces |
| K6 | Stir-fried vegetables |
| K7 | Broccoli, brussel sprouts, cabbage |
| K8 | Courgettes |
| K9 | Spinach |
| K10 | Parsnips |
| K11 | Radish |
| K12 | Leeks |
| K13 | Carrots |
| K14 | Cauliflower |
| K15 | Peas, sweetcorn |
| K16 | Celery |
| K17 | Peppers, red, green, yellow etc |
| K18 | Other vegetable |
| M1 | Fruit salad (tinned or fresh) |
| M2 | Apple |
| M3 | Pear |
| M4 | Banana |
| M5 | Orange, satsuma etc |
| M6 | Grapes |
| M7 | Melon, watermelon |
| M8 | Pineapple |
| M9 | Strawberry, raspberry etc |
| M10 | Peach, nectarine, plum, apricot, mango |
| M11 | Kiwi |
| M12 | Other fresh fruit |
| M13 | Dried fruit |
| **Meat, fish, eggs, beans and other non-dairy sources of protein** | |
| E4 | Quiche - meat, fish or vegetable |
| E5 | Scrambled egg, omelette, fried egg |
| E6 | Poached, boiled egg |
| F1 | Chicken, turkey……sliced or plain |
| F3 | Chicken, turkey ……in a creamy sauce, curry e.g. korma or tikka masala |
| G1 | Other meats……sliced roast, steak, chops |
| G2 | Other meats……stew casserole, mince, curry or keema |
| G9 | Offal e.g. liver, kidney |
| H3 | White fish (not fried) e.g. cod, haddock, plaice |
| H4 | Tuna or other oily fish (including can or fresh) |
| H5 | Shellfish e.g. prawns, mussels |
| K19 | Baked beans |
| K20 | Lentils, dahl |
| K21 | Other beans |
| I3 | Quorn, veggie mince, sausages etc |
| **Processed meat and fish products** | |
| F2 | Chicken, turkey…nuggets, dippers, kiev, etc |
| G3 | Other meats…beef burger, hamburger, doner, kebab |
| G4 | Bacon |
| G5 | Ham |
| G6 | Sausages |
| G7 | Sausage roll, meat pie, pasty, fried dumplings |
| G8 | Corned beef, luncheon meats, salami, pepperoni |
| H1 | Fish fingers |
| H2 | Fried fish in batter (as in fish and chips) |
| **Dairy** | |
| A1 | Milk, milky drink, lassi |
| B7 | Yoghurt, fromage frais |
| E1 | Hard cheese, e.g. Cheddar, Red Leicester |
| E2 | Cheese spread, triangle, string |
| E3 | Cottage cheese |
| N3 | Cream, custard |
| N4 | Mousse, milk puddings, e.g. rice pudding |
| P5 | Milk on cereal |
| **Desserts** | |
| B7 | Yogurt or fromage frais |
| M1 | Fruit salad (tinned or fresh) |
| N4 | Mousse, milk puddings, e.g. rice pudding |
| N2-3 and N5-6* | Ice cream, frozen dessert (e.g. Vienetta); Cream, custard; Cakes, buns, sponge pudding; Sweet pies, tarts, crumbles |
| *N2-3 and N5-6 should only be consumed as milk- or fruit-based desserts | |
| **High-sugar or high-fat snacks** | |
| B1 | Crisps, savoury snacks (cheddars) |
| B3 | Cereal bar, muesli bar, flapjack |
| B4 | Chocolate biscuit |
| B5 | Other biscuit |
| B6 | Croissant, waffles, pop tarts |
| N1 | Jelly, ice lolly |
| N2 | Ice cream, frozen dessert (e.g. Vienetta) |
| N3 | Cream, custard |
| N4 | Mousse, milk puddings, e.g. rice pudding |
| N5 | Cakes, buns, sponge pudding |
| N6 | Sweet pies, tarts, crumbles |
| O1 | Sweets, toffees, mints |
| O2 | Chocolate bars, e.g. Mars, Galaxy |
| *N2-6 should only be consumed during meal times as milk- or fruit-based desserts | |
| **Sugary drink** | |
| A3 | Drinking chocolate etc |
| A4 | Fizzy drink (pop/cola), squash, fruit drink (e.g. Ribena) |
| A5 | Diet, low calorie drink (including fizzy low calorie) |

**Supplementary Table 2: Food group intake or diet quality and odds of being overweight/obese^1^**

|  | **Healthy weight** | **Over-**  **weight/**  **obese** | **zBMI UK 1990, ORs and 95% CI** | | **Healthy weight** | **Over-weight/**  **obese** | **zBMI IOTF, ORs and 95% CI** | |
| --- | --- | --- | --- | --- | --- | --- | --- | --- |
| **Food groups** | **n** | **n** | **Crude** | **Adjusted^1^** | **n** | **n** | **Crude** | **Adjusted^1^** |
| Starchy foods | 100 | 40 | 1.24 (0.89, 1.73) | 1.16 (0.80, 1.69) | 99 | 35 | 1.15 (0.82, 1.61) | 1.05 (0.73, 1.50) |
| Fruit and vegetable | 100 | 40 | 0.98 (0.86, 1.12) | 0.97 (0.84, 1.11) | 99 | 35 | 0.99 (0.86, 1.13) | 0.98 (0.85, 1.12) |
| Meat, fish, eggs, beans, and non-dairy sources of protein | 100 | 40 | 0.71 (0.45, 1.13) | 0.73 (0.44, 1.20) | 99 | 35 | 0.70 (0.43, 1.13) | 0.72 (0.43, 1.19) |
| Milk and dairy foods | 100 | 40 | 1.15 (0.91, 1.47) | 1.15 (0.88, 1.49) | 99 | 35 | 1.11 (0.86, 1.43) | 1.12 (0.85, 1.47) |
| **NAPSACC Score** |  |  |  |  |  |  |  |  |
| One main meal | 44 | 19 | 0.90 (0.61, 1.34) | 0.78 (0.50, 1.23) | 46 | 16 | 1.03 (0.67, 1.56) | 0.91 (0.56, 1.48) |
| Two or more main meals | 47 | 18 | 0.95 (0.63, 1.42) | 0.92 (0.55, 1.55) | 45 | 16 | 0.89 (0.57, 1.38) | 0.87 (0.51, 1.49) |
| One snack | 52 | 21 | 0.93 (0.36,2.36) | 0.87 (0.28,2.72) | 52 | 18 | 0.86 (0.33,2.25) | 0.71 (0.24,2.10) |
| Two snacks | 32 | 14 | 2.39 (0.94,6.07) | 2.13 (0.63,7.23) | 34 | 12 | 3.81 (1.25,11.58) | 3.75 (0.99,14.22) |

ORs: Odds Ratios; CI: Confidence Intervals

^1^Adjusted for age, ethnicity, parental education and cluster; using healthy weight as the reference group

**Supplementary Table 3: Accelerometer physical activity and sedentary time by nursery and non-nursery day**

|  | **Nursery** | **Non-nursery** | **Adjusted mean difference^1^ (95% CI)** | **p-value** |
| --- | --- | --- | --- | --- |
|  | n = 113 | n = 104 |  |  |
| Counts per minute | 564.50 | 566.21 | 0.43 (-37.14, 37.99) | 0.982 |
| Minutes spent in MVPA | 22.54 | 21.50 | -1.16 (-3.80, 1.48) | 0.391 |
| Minutes spent in LPA | 124.50 | 115.40 | -9.34 (-16.75, -1.93) | 0.014 |
| Minutes spent in active time | 147.04 | 136.89 | -10.51 (-19.80, -1.22) | 0.027 |
| Minutes spent in sedentary | 504.56 | 481.07 | -22.84 (-39.64, -6.04) | 0.008 |
| Proportion of time spent in MVPA | 3.45 | 3.54 | 0.07 (-0.36, 0.50) | 0.742 |
| Proportion of time spent in LPA | 19.08 | 18.76 | -0.37 (-1.46, 0.72) | 0.504 |
| Proportion of time spent in active time | 22.53 | 22.30 | -0.30 (-1.71, 1.12) | 0.682 |
| Proportion of time spent in sedentary | 77.47 | 77.70 | 0.30 (-1.12, 1.71) | 0.682 |

^1^ Adjusted for gender, age, ethnicity, parental education and cluster; using nursery day as the reference group

**Supplementary Table 4: Accelerometer physical activity and sedentary time by gender on nursery and non-nursery day**

| **Nursery Day** | **Boys** | **Girls** | **Adjusted mean difference^1^ (95% CI)** | **p-value** |
| --- | --- | --- | --- | --- |
|  | **n = 60** | **n = 53** |  |  |
| Counts per minute | 589.70 | 535.98 | -57.30 (-111.87, -2.72) | 0.040 |
| Minutes spent in MVPA | 24.74 | 20.04 | -4.88 (-9.00, -0.76) | 0.020 |
| Minutes spent in LPA | 129.52 | 118.82 | -11.68 (-22.82, -0.54) | 0.040 |
| Minutes spent in active time | 154.26 | 138.85 | -16.56 (-30.89, -2.23) | 0.024 |
| Minutes spent in sedentary | 497.75 | 512.28 | 15.08 (-7.92, 38.08) | 0.199 |
| Proportion of time spent in MVPA | 3.77 | 3.09 | -0.71 (-1.33, -0.09) | 0.024 |
| Proportion of time spent in LPA | 19.80 | 18.27 | -1.66 (-3.25, -0.07) | 0.041 |
| Proportion of time spent in active time | 23.58 | 21.36 | -2.37 (-4.45, -0.30) | 0.025 |
| Proportion of time spent in sedentary | 76.42 | 78.64 | 2.37 (0.30, 4.45) | 0.025 |
| **Non-Nursery Day** | **n = 57** | **n = 47** |  |  |
| Counts per minute | 591.40 | 535.65 | -57.19 (-107.81, -6.57) | 0.027 |
| Minutes spent in MVPA | 23.14 | 19.50 | -3.45 (-6.69, -0.20) | 0.037 |
| Minutes spent in LPA | 119.00 | 111.03 | -8.03 (-17.20, 1.13) | 0.086 |
| Minutes spent in active time | 142.14 | 130.53 | -11.30 (-22.33, -0.26) | 0.045 |
| Minutes spent in sedentary | 477.15 | 485.84 | 10.81 (-14.15, 35.77) | 0.396 |
| Proportion of time spent in MVPA | 3.82 | 3.20 | -0.60 (-1.19, -0.01) | 0.046 |
| Proportion of time spent in LPA | 19.36 | 18.03 | -1.52 (-2.99, -0.06) | 0.042 |
| Proportion of time spent in active time | 23.17 | 21.23 | -2.12 (-3.99, -0.25) | 0.027 |
| Proportion of time spent in sedentary | 76.83 | 78.77 | 2.12 (0.25, 3.99) | 0.027 |

^1^Adjusted for age, ethnicity, parental education and cluster; using boys as the reference group

**Supplementary Table 5: Physical activity and odds of being overweight/obese^1^**

| **UK 1990 Growth Reference Charts^2^** | **Crude ORs (95% CI)** | **p-value** | **Adjusted ORs^3^ (95% CI)** | **p-value** |
| --- | --- | --- | --- | --- |
| Counts per minute | 1.002 (0.999, 1.005) | 0.216 | 1.005 (1.001, 1.009) | 0.015 |
| Minutes spent in MVPA | 1.016 (0.973, 1.061) | 0.468 | 1.059 (1.004, 1.118) | 0.036 |
| Minutes spent in LPA | 1.011 (0.995, 1.028) | 0.176 | 1.025 (1.005, 1.044) | 0.013 |
| Minutes spent in active time | 1.008 (0.996, 1.021) | 0.202 | 1.020 (1.005, 1.036) | 0.010 |
| Minutes spent in sedentary | 0.996 (0.987, 1.004) | 0.301 | 0.995 (0.986, 1.004) | 0.299 |
| Proportion of time spent in MVPA | 1.107 (0.841, 1.458) | 0.467 | 1.404 (1.000, 1.971) | 0.050 |
| Proportion of time spent in LPA | 1.082 (0.969, 1.208) | 0.162 | 1.162 (1.024, 1.320) | 0.020 |
| Proportion of time spent in active time | 1.058 (0.972, 1.152) | 0.192 | 1.130 (1.022, 1.250) | 0.017 |
| Proportion of time spent in sedentary | 0.945 (0.868, 1.029) | 0.192 | 0.885 (0.800, 0.978) | 0.017 |
| **IOTF Growth Reference Charts^2^** |  |  |  |  |
| Counts per minute | 1.002 (0.999, 1.006) | 0.191 | 1.005 (1.001, 1.009) | 0.013 |
| Minutes spent in MVPA | 1.012 (0.967, 1.060) | 0.607 | 1.055 (0.997, 1.117) | 0.063 |
| Minutes spent in LPA | 1.017 (0.999, 1.035) | 0.063 | 1.0301 (1.009, 1.051) | 0.004 |
| Minutes spent in active time | 1.011 (0.998, 1.025) | 0.105 | 1.023 (1.007, 1.040) | 0.005 |
| Minutes spent in sedentary | 0.997 (0.988, 1.006) | 0.499 | 0.996 (0.986, 1.006) | 0.412 |
| Proportion of time spent in MVPA | 1.070 (0.799, 1.433) | 0.651 | 1.358 (0.949, 1.943) | 0.094 |
| Proportion of time spent in LPA | 1.115 (0.990, 1.256) | 0.072 | 1.200 (1.048, 1.373) | 0.008 |
| Proportion of time spent in active time | 1.074 (0.981, 1.176) | 0.125 | 1.149 (1.033, 1.278) | 0.010 |
| Proportion of time spent in sedentary | 0.931 (0.851, 1.020) | 0.125 | 0.870 (0.782, 0.968) | 0.010 |

ORs: Odds ratios; CI: Confidence intervals

^1^ Using healthy weight as reference category

^2^ Sample sizes: UK 1990 (n=112), and IOTF (n=109)

^3^ Adjusted for gender, age, ethnicity, parental education and cluster
